# Impact of PYCNOGENOL and Pomegranate Extract on Pigmentation and Lightness in Skin of Color: A Pilot Study

**DOI:** 10.1111/jocd.70757

**Published:** 2026-03-17

**Authors:** Sivavallinathan Arunachalam

**Affiliations:** ^1^ Render Skin and Hair Chennai India

To the Editor,

Hyperpigmentation is among the most frequent concerns in individuals with skin of color, often presenting as melasma or post‐inflammatory hyperpigmentation. Conventional treatment relies on topical depigmenting agents, such as hydroquinone or chemical peels, which may be limited by irritation, recurrence, or safety concerns with long‐term use. Plant‐derived systemic agents are therefore of increasing interest for safe pigment modulation. Pycnogenol, a standardized French maritime pine bark extract, and pomegranate extract are notable for their antioxidant and photoprotective properties and have shown promise in regulating melanogenesis [1, 2, 3].

We conducted a single‐arm, four‐week pilot study to assess the effect of a novel nutraceutical containing Pycnogenol and pomegranate extract (with 40% Punicalgins) on pigmentation outcomes in Indian adults. Ten healthy volunteers (aged 18–45 years) consumed one capsule daily (50 mg Pycnogenol, 187.5 mg pomegranate extract). Skin parameters were assessed using instruments from Courage + Khazaka Electronic GmbH (Germany): Mexameter (MX 18) with a probe area of 19.6 mm^2^ for melanin quantification, Corneometer (CM 825) with 49 mm^2^ area and small penetration depth of 10–20 μm for stratum corneum hydration, and Skin‐Colorimeter systems (13 mm/7 mm Ø for Flex CL 440 and 5 mm Ø for CL 400) for CIE L*a*b* color space. Calibration was conducted monthly for the Mexameter and Corneometer, and daily for the Colorimeter, following internal device‐specific procedures. Measurements were performed at a controlled ambient temperature of 25°C, relative humidity of 30%–50% and a constant UV index of 5uv [4]. Measurements for each site was taken once each for Mexameter, Spectrophotometer, Colorimeter and thrice for Corneometer. Safety was monitored through adverse event reporting. Ethics approval was obtained, and participants provided written informed consent.

## 1 Results

Paired *t*‐test was performed and significant reductions in melanin index were observed, alongside increases in skin lightness at the end of Day 28 (Table 1). The average improvements in L* values for the face and the forearm were 3.8 and 2.7, respectively. Hydration changes were minimal and not statistically significant (*p* > 0.05). No adverse events were reported, and tolerability was excellent among all participants.

**TABLE 1:** Changes in pigmentation and lightness indices after 4 weeks of Pycnogenol and pomegranate supplementation.

Parameter	Anatomical site	Baseline (mean ± SD)	Day 28 (mean ± SD)	% change in mean value	*p* (< 0.05—significant)
Melanin index	Forearm (*n* = 10)	448.4 ± 86.1	405.8 ± 52.5	−9.5%	0.01552
	Face (*n* = 10)	365.6 ± 75.1	343.4 ± 65.7	−6.1%	0.000924
Skin lightness (L* index)	Forearm (*n* = 10)	37.5 ± 2.4	40.2 ± 2.8	+7.1%	0.00053
	Face (*n* = 10)	37.1 ± 4.4	40.9 ± 2.8	+10.3%	0.007857

## 2 Discussion

The findings demonstrate that the studied nutraceutical agent containing Pycnogenol and pomegranate extract significantly reduced skin melanin index and improved the lightness index within 4 weeks. A ΔL* of > 2, considered clinically perceptible [5], was achieved for readings from both the face and the forearm.

The active agents in the nutraceutical, Pycnogenol and pomegranate extracts are documented tyrosinase inhibitors. Additionally, both agents also promote dermal health which has recently been shown to positively impact epidermal pigmentation. Pycnogenol down‐regulates select matrix metalloproteinases and reduces collagen fragmentation, while pomegranate offers broader suppression of degradative and inflammatory enzymes such as collagenase, elastase, and lipase. The synergistic effects of these plant‐derived actives support the role of this novel nutraceutical agent as a systemic adjunct for hyperpigmentation [3, 6].

Limitations include the small cohort, lack of control group, and short follow‐up; larger and longer studies would be helpful to establish the efficacy of this nutraceutical in specific pigmentary conditions for skin of color.

## 3 Conclusion

We observed that supplementation with this novel nutraceutical agent—combining Pycnogenol and pomegranate extract—was associated with reductions in melanin index and improvements in skin lightness within 4 weeks, with no safety concerns in this cohort. While preliminary, these findings suggest that this combined plant‐derived formulation may hold potential as a safe systemic adjunct in the management of hyperpigmentation in skin of color.

## Funding

The author has nothing to report.

## Ethics Statement

This study was undertaken by Mittal Global Clinical Trial Services (MGCTS) on behalf of Silphion Research Private Limited, the study sponsor. MGCTS was responsible for study execution, monitoring, and data management in accordance with Good Clinical Practice (GCP) guidelines, the Declaration of Helsinki (2013), and applicable national regulatory standards. Silphion Research Private Limited retained overall scientific and ethical oversight of the study, including adherence to Institutional Ethics Committee (IEC) approval requirements. Ethical approval for this study was obtained from the Institutional Ethics Committee of AR Multispecialty Hospital & Research Centre, Meerut dated 8th May 2024. All study participants were informed in detail about the nature, objectives, potential benefits, and possible risks of the study prior to enrolment and written informed consent was obtained from each participant with assurance of confidentiality, their voluntary right to withdraw at any time without penalty, and the use of collected data solely for research purposes.

## Conflicts of Interest

The author declares no conflicts of interest.

## Data Availability

The data that support the findings of this study are available on request from the corresponding author. The data are not publicly available due to privacy or ethical restrictions.
